# Long non-coding RNA SNHG15 inhibits P15 and KLF2 expression to promote pancreatic cancer proliferation through EZH2-mediated H3K27me3

**DOI:** 10.18632/oncotarget.20359

**Published:** 2017-08-18

**Authors:** Zhonghua Ma, Hesuyuan Huang, Jirong Wang, Yan Zhou, Fuxing Pu, Qinghong Zhao, Peng Peng, Bingqing Hui, Hao Ji, Keming Wang

**Affiliations:** ^1^ The Second Clinical Medical College of Nanjing Medical University, Nanjing 210000, Jiangsu, People's Republic of China; ^2^ Department of Oncology, Second Affiliated Hospital, Nanjing Medical University, Nanjing 210000, Jiangsu, People's Republic of China; ^3^ Department of Cardiothoracic Surgery, Children's Hospital of Nanjing Medical University, Nanjing 210008, Jiangsu, People's Republic of China; ^4^ Department of Oncology, The Affiliated Yixing Hospital of Jiangsu University, Wuxi 214200, Jiangsu, People's Republic of China; ^5^ Department of Medical Center for Digestive Diseases, Second Affiliated Hospital, Nanjing Medical University, Nanjing 210000, Jiangsu, People's Republic of China; ^6^ Department of General Surgery, Second Affiliated Hospital, Nanjing Medical University, Nanjing 210000, Jiangsu, People's Republic of China; ^7^ Department of Oncology, Second Hospital of Nanjing, Nanjing 210000, Jiangsu, People's Republic of China

**Keywords:** long noncoding RNA, SNHG15, pancreatic cancer, proliferation, P15 and KLF2

## Abstract

Long non-coding RNA (lncRNA) is emerging as an critical regulator in multiple cancers, including pancreatic cancer (PC). Recently, lncRNA SNHG15 was found to be up-regulated in gastric cancer and hepatocellular carcinoma, exerting oncogenic effects. Nevertheless, the biological function and regulatory mechanism of SNHG15 remain unclear in pancreatic cancer (PC). In this study, we reported that SNHG15 expression was also upregulated in PC tissues, and its overexpression was remarkably associated with tumor size, tumor node metastasis (TNM) stage and lymph node metastasis in patients with PC. SNHG15 knockdown inhibited proliferative capacities and suppressed apoptotic rate of PC cells *in vitro*, and impaired *in-vivo* tumorigenicity. Additionally, RNA immunoprecipitation (RIP) assays showed that SNHG15 epigenetically repressed the P15 and Kruppel-like factor 2 (KLF2) expression via binding to enhancer of zeste homolog 2 (EZH2), and chromatin immunoprecipitation assays (CHIP) assays demonstrated that EZH2 was capable of binding to promoter regions of P15 and KLF2 to induce histone H3 lysine 27 trimethylation (H3K27me3). Furthermore, rescue experiments indicated that SNHG15 oncogenic function partially involved P15 and KLF2 repression. Consistently, an inverse correlation between the expression of SNHG15 and traget genes were found in PC tissues. Our results reported that SNHG15 could act as an oncogene in PC, revealing its potential value as a biomarker for early detection and individualized therapy.

## INTRODUCTION

Pancreatic cancer (PC) is now one of the leading causes of cancer-related death, especially in the United States, with an estimation of 43,090 deaths in 2017 [1]. Compared with the steadily increasing survival rates in some cancers, colorectal cancer is characterized by a poor prognosis, which limits the 5-year relative survival to only 8 % [1]. The adverse outcome is largely due to the inability to diagnose PC in its early stages, with ~53% of patients expreiencing metastasis at the time of diagnosis [2]. Therefore, there is a critical need to increase the understanding of the underlying mechanisms associated with PC.

Current advances in bioinformatics and sequencing technology have led to the discovery of long non-coding RNAs (lncRNAs) [3, 4]. lncRNAs were once regarded as transcriptional “noise” due to their lack of protein-encoding capability [5]; however, emerging evidence suggests that lncRNAs participate in multiple biological processes, including imprinting, epigenetic regulation, alternative splicing, RNA decay, cell differentiation, cell cycle control, and cancer-cell metastasis [6–8]. Furthermore, lncRNAs are dysregulated in many cancers [9, 10], and aberrant lncRNA expressions is significantly associated with carcinogenesis [11]. LncRNA may act as an oncogene [12, 13] or a tumor suppressor [14], and have potential as a cancer biomarker [15, 16]. LncRNA HOX transcript antisense RNA (HOTAIR) has been identified as an unfavourable prognostic indicator in patients with breast, liver, colon, and pancreatic cancer [17–21]. Additionally, we previously reported that lncRNA SPRY4-IT1 overexpression correlated with poor outcomes of patients with breast cancer and could promote breast cancer proliferation [22]. LncRNA HOXA transcript at the distal tip (HOTTIP) can mediate colorectal cancer (CRC) cell proliferative capacities by downregulating p21 expression [23]. Therefore, lncRNAs may serve as novel biomarkers for cancer diagnosis and molecular therapy.

Recently, lncRNAs dysregulations were reportedly involved in PC development and progression [24–26]. Elevtaed expression of long intergenic non-coding RNA ROR (lincRNA-ROR) and lncRNA nuclear paraspeckle assembly transcript 1 (NEAT1) promotes PC cell proliferation, invasion, and metastasis [27–30]. Furthermore, several lncRNAs, such as metastasis-associated lung adenocarcinoma transcription 1 (MALAT1), HOTTIP, and HOTAIR, have been characterized as negative prognostic factors in PC patients, indicating that they might exert pro-oncogenic roles *in vitro* and *in vivo* [10, 16, 19, 26, 31]. Our previous studies revealed that lncRNA IRAIN could inhibit PC cell apoptosis and increase its proliferative capacities with interaction with polycomb repressive complex 2 (PRC2) [32]. These results revealed the critical functions of lncRNAs in PC pathogenesis, highlighting the importance of further investigation and identification of lncRNAs.

The lncRNA SNHG15 is 837bp in length, and located on chromosome 7p13 (https://www.ncbi.nlm.nih.gov/nuccore/NR_003697.1). It was firstly reported to exhibit significant upregulation in gastric cancer (GC) tissue samples and cell lines. Functional studies suggested the involvement of SNHG15 in GC cell proliferation and invasion [33]. Moreover, lncRNA SNHG15 was found to be associated with histological grade, tumor node metastasis stage (TNM) stage, and poor overall survival in hepatocellular carcinoma (HCC), suggesting its potential role as a novel biomarker in HCC patients [34]. However, the expression pattern, functional role and underlying mechanism of SNHG15 are completely unknown in PC. According to previous reports, we observed that pro-oncogenic lncRNAs exhibit significant upregulation in PC tissues and cell lines, with their aberrant expressions potentially influencing cancer cell growth, survival and migration/invasion. Furthermore, HOTAIR knockdown in PC cells could alter cell cycle, impair cell proliferation, and promote apoptosis *in vitro*, and inhibit tumorigenesis abilities *in vivo* [19].

Here, we report, for the first time, the expression pattern, function and regulatory mechanism of SNHG15 in PC. Quantitative reverse transcription polymerase chain reaction (qRT-PCR) assays demonstrated that SNHG15 expression was significantly increased in PC tissue samples and cell lines, suggesting pro-oncogenic functions similar to those reported for HOTAIR. Furthermore, our findings indicated that lncRNA SNHG15 promoted pancreatic cancer cell proliferation through epigenetic repression of P15 and Kruppel-like factor 2 (KLF2). Although SNHG15 and HOTAIR exhibit similar pro-oncogenic roles, our findings suggested that the downstream targets and regulatory pathways associated with both lncRNAs differed.

## RESULTS

### LncRNA SNHG15 is increased in PC tissues, and significantly associated with tumor size, TNM stage, and lymph node metastasis in patients with PC

**Figure 1:**
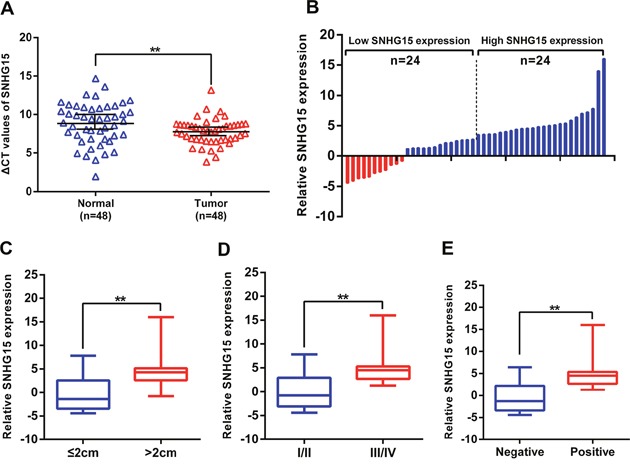
SNHG15 expression is upregulated in PC tissues and its clinical significance **(A)** Relative expression of SNHG15 in human PC tissues (n=48) compared with corresponding adjacent normal tissues (n=48). SNHG15 expression was examined by qPCR and normalized to GAPDH expression (shown as ΔCT). **(B)** The patients were classified into two groups according to SNHG15 expression. **(C-E)** The results are presented as relative expression levels in tumor tissues. SNHG15 expression was significantly higher in patients with a larger tumor size, a higher pathological stage, and lymph node metastasis (shown as ΔCT). Bars: s.d, ^*^P<0.05, ^**^P<0.01.

We analyzed the expression of SNHG15 in a cohort of 48 PC tissue samples and matched non-tumor samples using qRT-PCR analysis, with our resuts showing that SNHG15 was remarkly increased in PC tissue samples relative to levels observed in adjacent normal tissues (Figure 1A). To study the correlation between SNHG15 levels and the clinicopathologic characteristics of PC patients, we classified 48 PC patients into two groups: the high (n=24, fold change ≥ median value) and the low SNHG15 group (n=24, fold change ≤ median value) based on the median value of SNHG15 expression (Figure 1B). We observed that tumor size (*p* = 0.017), TNM stage (*p* = 0.009), and lymph node metastasis (*p* = 0.001) were positively associated with increased SNHG15 expression (Figure 1C–1E). As shown in Table 1, no significant relationships were found between increased SNHG15 expression and other clinicopathologic factors, such as gender (*p* = 0.771) and age (*p* = 0.562). These findings indicate that SNHG15 is an unfavourable factor for PC patients and have potential as a novel biomarker for PC patients.

**Table 1:** Correlations between SNHG15 expression and clinicopathological characteristics of PC patients (n=48)

Characteristics	SNHG15 expression		P Chi-squared test P-value
	Low	High	
**Age(years)**			
>60	12	14	0.562
≤60	12	10	
**Gender**			
Male	13	14	0.771
Female	11	10	
**Differentiation**			
Well/moderate	10	7	0.365
Poor	14	17	
**Tumor size**			
≤2cm	13	5	0.017^*^
>2cm	11	19	
**TNM Stage**			
I/II	15	6	0.009^**^
III/IV	9	18	
**Lymph node metastasis**			
Positive	9	20	0.001^**^
Negative	15	4	

^*^P < 0.05, ^**^P < 0.01.

### Manipulation of SNHG15 expression in PC cells

**Figure 2:**
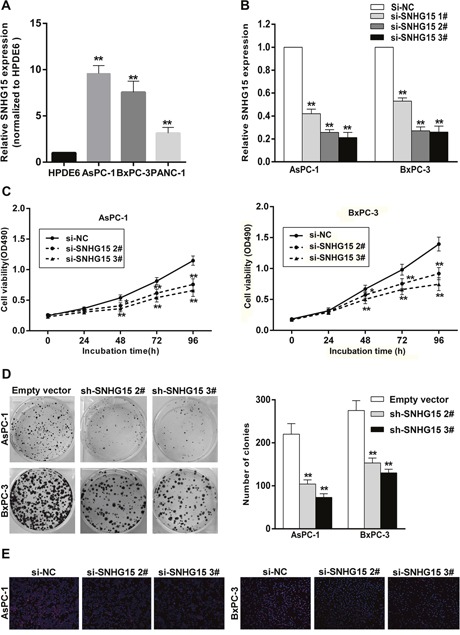
SNHG15 knockdown inhibits PC cell proliferation *in vitro* **(A)** SNHG15 expression levels of PC cell lines (AsPC-1, BxPC-3 and PANC-1), compared with that in human pancreatic ductal epithelial cells (HPDE6). **(B)** qRT-PCR analysis of SNHG15 expression in AsPC-1 and BxPC-3 cell lines transfected with SNHG15 siRNAs or the negative control. **(C)** MTT assays were performed to detect the viability of si-SNHG15 transfected AsPC-1 and BxPC-3 cells. **(D)** Colony-forming growth assays were performed to determine the proliferation of PC cells. The colonies were counted and captured. **(E)** Proliferating AsPC-1 and BxPC-3 cells were labeled with Edu. The Click-it reaction revealed Edu staining (red). Cell nuclei were stained with DAPI (blue). The images are representative of the results obtained. ^*^P<0.05 and ^**^P<0.01.

To test SNHG15 expression levels in PC cells, we performed qPCR assays and found that the expression levels of SNHG15 was upregulated in PC cell lines compared with that of the normal human pancreatic ductal epithelial cell (HPDE6). In this study, we select AsPC-1 and BxPC-3 cells due to their higher expression among three PC cell lines (Figure 2A). Then, SNHG15 expression was knocked down in AsPC-1 and BxPC-3 cells by transfection with small interfering RNAs (siRNAs) or a short hairpin RNA (shRNA) vector and overexpressed by transfection with a pcDNA-SNHG15 vector. qPCR analysis was performed at 48-h post-transfection, with the data revealing that SNHG15 expression was significantly reduced by siRNA-SNHG15 infection as compared with results observed in control cells. Furthermore, transfection with si-SNHG15 2# and si-SNHG15 3# exhibited more efficient interference relative to that observed with si-SNG15 1# transfection (Figure 2B); therefore, we selected the si-SNHG15 2# and si-SNG15 3# for use in the subsequent experiments. qPCR assays were also used to test SNHG15 expression in pcDNA-SNHG15-transfected AsPC-1 and BxPC-3 cells. Compared with the negative control, SNHG15 expression exhibited a remarkable increase in AsPC-1 and BxPC-3 cells following pcDNA-SNHG15 transfection (Supplementary Figure 1A).

### The effect of SNHG15-mediated proliferation in PC cells

To identify the function of SNHG15 in PC, we performed loss-of-function and gain-of-function assays. MTT assays showed that growth of AsPC-1 and BxPC-3 cells transfected with si-SNHG15 2# and si-SNHG15 3# was inhibited relative to control cells (Figure 2C), whereas SNHG15 overexpression promoted AsPC-1 and BxPC-3 cells proliferation (Supplementary Figure 1B). Similarly, decreased SNHG15 impaired colony-formation capacities of PC cells (Figure 2D), whereas SNHG15 overexpression increased AsPC-1 and BxPC-3 colon formation (Supplementary Figure 1C). These findings were confirmed by results of EdU staining assays (Figure 2E), and highlighted SNHG15 as an oncogene in PC cell lines.

### SNHG15 downregulation induces PC cell apoptosis and alters cell cycle progression *in vitro*

**Figure 3:**
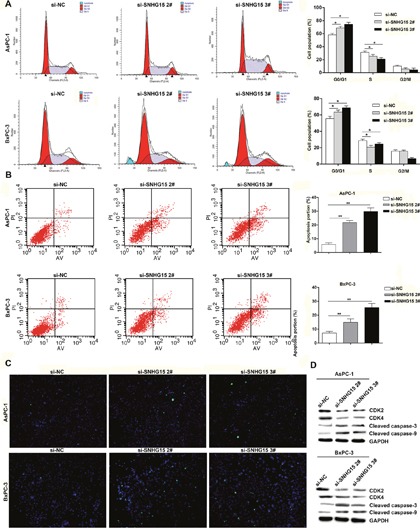
Knockdown of SNHG15 promotes cell cycle arrest and induces apoptosis in PC cells *in vitro* **(A)** Flow cytometry assays were performed to analysis the cell cycle progression when PC cells transfected with si-SNHG15. The bar chart represented the percentage of cells in G0/G1, S, or G2/M phase, as indicated. All experiments were performed in biological triplicates with three technical replicates. **(B)** Flow cytometry was used to detect the apoptotic rates of cells. LR, early apoptotic cells; UR, terminal apoptotic cells. **(C)** Apoptosis in AsPC-1 and BxPC-3 cells after SNHG15 knockdown was detected through TUNEL staining. **(D)** Western blot analysis of CDK2, CDK4 and cleaved caspase-3 and cleaved caspase-9 after si-NC, si-SNHG15 2#, or si-SNHG15 3# transfection in AsPC-1 and BxPC-3 cells. GAPDH protein was used as an internal control.

The involvement of cell cycle and apoptosis is required for regulating cell proliferative abilities. To evaluate SNHG15-mediated impact on PC cell cycle and apoptosis, flow cytometric assays and terminal deoxynucleotidyl transferase dUTP nick-end labeling (TUNEL) staining analysis were conducted. We observed a significant increase at G1/G0 phase in AsPC-1 or BxPC-3 cells transfected with si-SNHG15 2# and si-SNHG15 3#, compared with respective controls (Figure 3A). Moreover, AsPC-1 and BxPC-3 cells transfected with SNHG15 siRNAs exhibited higher levels of apoptosis as compared with control cells (Figure 3B-3C). Additionally, western blot assays found remarkable alteration of CDK2 (cyclin-dependent kinase 2) and CDK4 (cyclin-dependent kinase 4) in AsPC-1 and BxPC-3 cells of SNHG15 knockdown, confirming that SNHG15 involvement in cell cycle regulation (Figure 3D). Furthermore, protein expression levels of cleaved caspase-3 and cleaved caspase-9 genes exhibited significant increases in AsPC-1 and BxPC-3 transfected with SNHG15 siRNAs (Figure 3D). These data suggest that SNHG15 knockdown could promote G1/G0 arrest and increase apoptotic rate in PC cells *in vitro*.

### SNHG15 knockdown inhibits PC cell tumorigenesis *in vivo*

**Figure 4:**
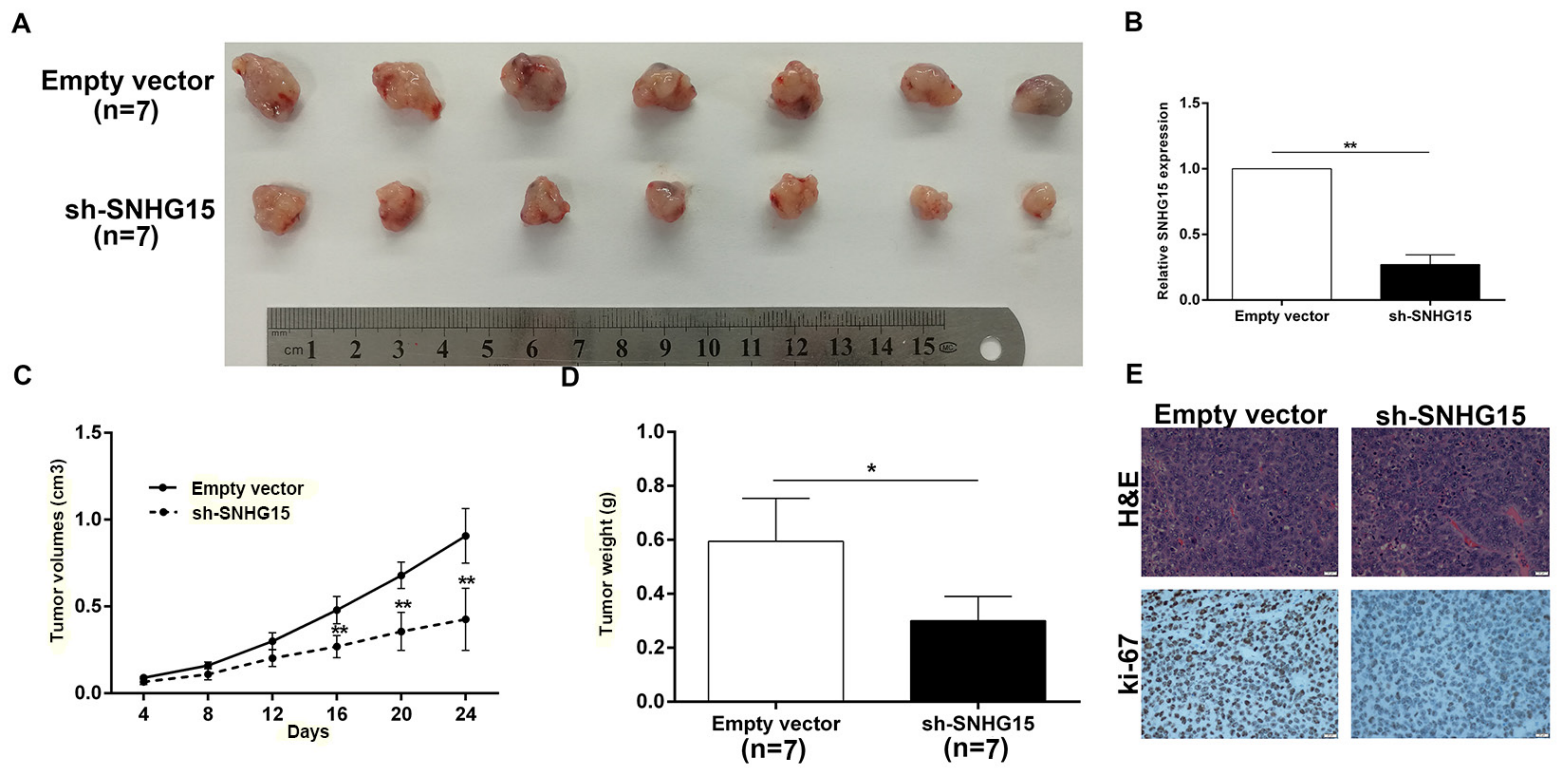
Knockdown of SNHG15 inhibits PC cell tumorigenesis *in vivo* **(A)** Empty vector or sh-SNHG15 were transfected into BxPC-3 cells, which were injected in the nude mice (n = 7), respectively. Tumors formed in sh-SNHG15 group were dramatically smaller than the control group. **(B)** qRT-PCR was performed to detect the average expression of SNHG15 in xenograft tumors (n = 7). **(C)** Tumor volumes were calculated after injection every four days. Points, mean (n = 7); bars indicate SD. **(D)** Tumor weights were represented as means of tumor weights±SD. **(E)** The tumor sections were under H&E staining and IHC staining using antibodies against ki-67. Error bars indicate mean ± standard errors of the mean. ^*^P < 0.05, ^**^P < 0.01.

To investigate the impact of SNHG15 *in vivo*, we inoculated empty vector or sh-SNHG15-transfected BxPC-3 cells into nude mice. Compared with control, the tumor derived from sh-SNHG15-transfected BxPC-3 cells was obviously smaller (Figure 4A, 4C). Consistently, the tumor weight from sh-SNHG15 group was remarkably lighter than control group (Figure 4D). Then, qPCR experiments determined an obvious decrease of SNHG15 in the tumor tissues derived from sh-SNHG15-transfected BxPC-3 cells, compared with that of respective control groups (Figure 4B). Immunohistochemistry (IHC) results found that the tumor tissues derived from sh-SNHG15-transfected BxPC-3 cells displayed lower Ki-67 staining than those formed from the control cells (Figure 4E). These findings indicated that SNHG15 downregulation suppressed PC cells tumor growth *in vivo*.

### SNHG15 epigenetically silences P15 and KLF2 transcription by binding to EZH2

Mounting studies indicated that lncRNA was capable of regulating target-gene expression through interactions with RNA binding proteins [10, 35–39]. For example, lincRNA00511 suppresses p57 expression via interaction with EZH2 in non-small-cell lung carcinoma (NSCLC). LncRNA SNHG15 was initially found to be overexpressed in GC and mediated GC cell proliferation and invasion [33]; however, the molecular mechanism and downstream targets of SNHG15 involved in PC cell proliferation remains unknown.

**Figure 5:**
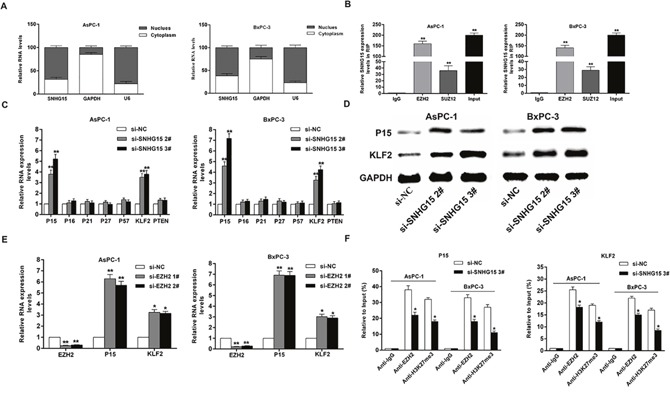
SNHG15 epigenetically silences P15 and KLF2 transcription by binding to EZH2 **(A)** qRT-PCR analysis of SNHG15 nuclear and cytoplasmic expression levels in AsPC-1 and BxPC-3 cells. U6 was used as a nucleus marker, and GAPDH was used as a cytosol marker. **(B)** RIP experiments were performed in AsPC-1 and BxPC-3 cells, and the coprecipitated RNA was subjected to qRT-PCR for SNHG15. The fold enrichment of SNHG15 in EZH2/SUZ12 RIP is relative to its matched IgG control. **(C)** The levels of p15, p16, p21, p27, p57, KLF2 and PTEN mRNA were determined by qRT–PCR when knockdown of SNHG15. **(D)** The p15 and KLF2 protein levels were determined by western blot in SNHG15 knockdown in AsPC-1 and BxPC-3 cells. **(E)** The p15 and KLF2 expression levels were determined by qRT-PCR in AsPC-1 and BxPC-3 cells transfected with si-EZH2 1# or 2#. **(F)** ChIP-qRT-PCR of EZH2 occupancy and H3K27me3 binding in the p15 and KLF2 promoters in AsPC-1 and BxPC-3 cells treated with si-SNHG15 3# (48 h) or si-NC; IgG as a negative control. Error bars indicate mean ± standard errors of the mean. ^*^P < 0.05, ^**^P < 0.01.

To investigate the regulatory mechanism of SNHG15, we performed subcellular fractionation assays and found that SNHG15 expression was much higher in nucleus than cytoplasm (Figure 5A). Then, RIP experiment confirmed that SNHG15 interacted with EZH2 in AsPC-1 and BxPC-3 cells (Figure 5B). Similarly, endogenous SNHG15 exhibited enrichment in anti-SUZ12 RIP fraction. Thus, SNHG15 may downregulate the expression of target genes through binding to PRC2 at epigenetical levels.

To discover key downstream targets of SNHG15, we selected potential EZH2 and SUZ12 targets and determined their involvement in SNHG15-related PC development. Result of qPCR assays showed that P15 and KLF2 expression levels were elevated in AsPC-1 and BxPC-3 cells following transfection of si-SNHG15 2# and si-SNHG15 3#; however, there were no significant differences in the expressions of other genes following SNHG15 knockdown (Figure 5C). Additionally, the protein levels of P15 and KLF2 exhibited significant alterations in si-SNHG15-treated cells (Figure 5D). Furthermore, AsPC-1 and BxPC-3 cells transfected with EZH2 siRNAs effectively decreased EZH2 expression and an obvious increase of P15 and KLF2 expression was observed (Figure 5E). Consistently, SUZ12 inhibition leads to upregulation of P15 and KLF2 in AsPC-1 and BxPC-3 cells (Supplementary Figure 2). Our findings showed that P15 and KLF2 were the most up-regulated mRNAs in SNHG15-depleted PC cells, EZH2-depleted PC cells, and SUZ12-depleted PC cells. These results suggested that P15 and KLF2 may be novel downstream targets of SNHG15.

To determine whether SNHG15 suppresses the expression of P15 and KLF2 by binding to EZH2, we performed chromatin immunoprecipitation (CHIP) assays. The results found that EZH2 bound the promoter regions of P15 and KLF2, and mediated histone H3 lysine 27 trimethylation (H3K27me3) modification. However, SNHG15 knockdown reduced this binding activity and H3K27me3 levels (Figure 5F). These results illustrated that SNHG15 could regulate the expression of P15 and KLF2 partially via interaction with EZH2 in PC cells.

### P15 and KLF2 involvement in SNHG15-mediated oncogenic role

**Figure 6:**
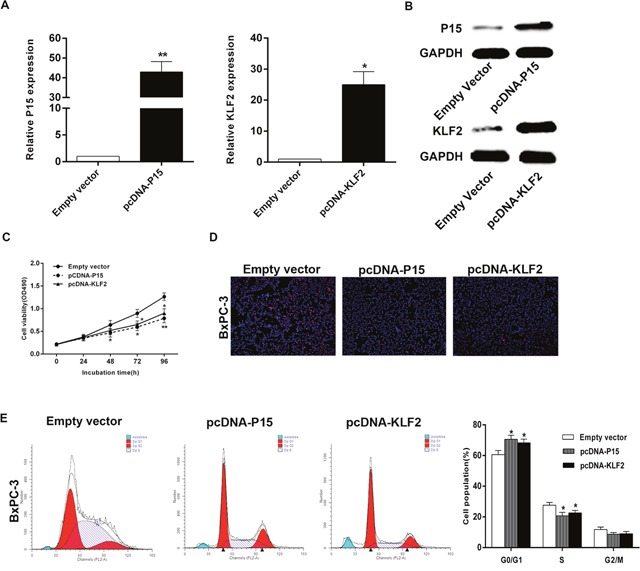
Effect of P15 and KLF2 of overexpression on BxPC-3 cell *in vitro* **(A, B)** The mRNA levels and protein levels of P15 and KLF2 in BxPC-3 cells transfected with pCDNA-P15 or pCDNA-KLF2 was detected by qPCR analysis. **(C, D)** MTT assays and Edu staining assays were used to determine the cell viability. Values represent the mean ± s.d. from three independent experiments. **(E)** Cell cycle was analyzed by flow cytometry. The bar chart represents the percentage of cells in G1–G0, S, or G2–M phase, as indicated. ^*^P < 0.05 and ^**^P < 0.01.

Gain-of-function analysis was conducted to study P15 and KLF2 involvement in SNHG15-mediated PC cell proliferation. Compared with control group, the protein levels of P15 and KLF2 were found to be upregulated in BxPC-3 cells following transfection with pcDNA-P15 and pcDNA-KLF2 (Figure 6A, 6B). Additionally, MTT results demonstrated that cell proliferation was inhibited upon P15 and KLF2 overexpression, and EdU assays showed the same results (Figure 6C, 6D). Furthermore, we found that aberrant expression of P15 and KLF2 also induced G1/G0 phase arrest (Figure 6E). These findings indicated that SNHG15 exhibited oncogenic effects in PC cells partially through repression of P15 and KLF2 expression.

**Figure 7:**
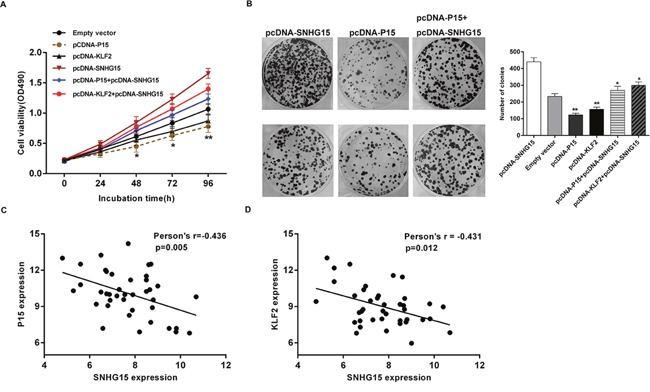
SNHG15 negatively regulates expression of P15 and KLF2 by rescue assays **(A, B)** MTT and colony formation assays were used to determine the cell proliferation ability for BxPC-3 cells transfected with pCDNA-SNHG15 and pCDNA-P15 and pCDNA-KLF2 and co-transfected with pCDNA-SNHG15 and pCDNA-P15 or pCDNA-SNHG15 and pCDNA-KLF2. **(C)** qPCR analyzed the P15 and KLF2 mRNA levels in 40 pairs PC tissues and found that there was a significantly negative correlation between SNHG15 and P15 or KLF2. Values represent the mean±s.d. from three independent experiments.

Moreover, we conducted rescue assays to determine P15 and KLF2 involvement in SNHG15 contributions to PC cell proliferation. BxPC-3 cells were co-transfected with pcDNA-SNHG15 and pCNDA-P15 or pCDNA-KLF2. We observed that pcDNA-P15 or pcDNA-KLF2 transfection partially rescue pcDNA-SNHG15-transfection-mediated decreases in P15 or KLF2 expression, and the co-transfection could partly reverse pcDNA-SNHG15-induced growth (Figure 7A-7D). Furthermore, we detected correlations between SNHG15 and P15 and KLF2 expression in 40 pairs of PC tissues, revealing a significantly negative correlation between SNHG15 and P15 or KLF2 expression (Figure 7E). These findings indicated that SNHG15 exhibited oncogenic effects in PC cells partially through repression of P15 and KLF2 expression.

## DISCUSSION

With advances in sequencing technologies, hundreds of lncRNAs in human cancers have been discovered. Current evidence has highlighted lncRNAs as crucial modulators and key players in multiple malignancies, including PC [12, 29, 40, 41]. LncRNA SPRY4-IT1 increases proliferative abilities of breast cancer cells through upregulation of zinc finger protein 703 (ZNF703) expression [22]. Additionally, lncRNA growth-arrest-specific 5 (GAS5) plays tumor-suppressive roles in PC cells [42], whereas NEAT1, MALAT1, HOTTIP and HOTAIR exert oncogenic roles in PC cells [26]. Our previous study also reported that lncRNA IRAIN could serve as an oncogene in PC cells and promote proliferation through silencing P15 and KLF2 [32].

A growing body of evidence suggests that lncRNA can silence downstream target expression through binding to RNA-binding proteins (RBPs) or competing for binding to microRNAs [10, 35–37]. Sun et al. [43] reported that lncRNA HOXA11-AS could interact with EZH2, LSD1 and DNA methyltransferase 1 (DNMT1) to exert oncogenic functions in GC. Additionally, ~20% of lncRNAs are capable of binding to PRC2 to regulate target gene expression [44]. LncRNA HOTAIR interacts with PRC2 to induce H3K27me3, thus silencing the expressions of downstream targets [19, 45]. Understanding HOTAIR activity helps elucidate the mechanism associated with lncRNA binding to PRC2, resulting in transcriptional repression through direct silencing of specific loci. EZH2, a catalytic component of PRC2 [46], can induce H3K27me3 to repress gene transcription with specificity [47, 48]. More importantly, EZH2 is involved in multiple pathological processes related to carcinogenesis, proliferation, apoptosis and metastasis [49–51]. Kyounghyun et al. [10, 19] found that decreased HOTAIR expression enriched expression of cell-cycle-related genes, and that HOTAIR activities in PC are partly dependent on interaction of HOTAIR with EZH2, which induces H3K27me3 to silence expressions of multiple genes.

HOTAIR has been reported to exert pro-oncogenic functions in many cancers, including PC [19, 45, 52–55] and is significantly overexpressed in PC tissues, with HOTAIR knockdown impairing cell growth, blocking cell cycle progression, and inducing apoptosis in PC cells [19]. Additionally, gene-set-enrichment analysis (GSEA) analysis revealed a critical role for HOTAIR in cell cycle progression and proliferation. In this study, we investigated SNHG15 expression and function in PC cell lines. *In-vitro* assays revealed that decreased SNHG15 inhibited PC cell proliferative capacities, promoted G1/G0 phase arrest, and activated apoptosis, whereas *in-vivo* assay demonstrated that SNHG15 downregulation suppressed PC cell tumorigenesis. SNHG15 exhibits functions similar to those reported for HOTAIR and exerts pro-oncogenic roles in PC progression; however, the downstream targets and regulatory pathways differ between HOTAIR and SNHG15. HOTAIR-mediated suppression of genes in PC is partly EZH2-dependent [10, 19], and HOTAIR knockdown affects genes involved in cell cycle progression and proliferation, including growth/differentiation factor 15 (GDF15), which inhibits cell growth and activates apoptosis [56, 57] and is co-regulated by HOTAIR and EZH2 [19].

In this study, we reported that SNHG15 could promote PC cell proliferation by interacting with EZH2, but the genes mediated by HOTAIR or SNHG15 are different. The tumor suppressors P15 and KLF2 were the most highly upregulated genes in SNHG15-depleted PC cells, EZH2-depleted PC cells, and SUZ12-depleted PC cells. Chip assays confirmed that SNHG15 can recruit EZH2 to P15 and KLF2 promoter regions and represses transcriptions of P15 and KLF2 through H3K27me3 modification in PC cells. To our knowledge, there is no report concerning the regulatory mechanism between HOTAIR and P15 or KLF2 now. Thus, although SNHG15 and HOTAIR have similar functions in pancreatic cancer, they differ in terms of target genes and their mechanisms of action.

Cyclin-dependent protein kinase inhibitors (CKIs) regulate cell cycle progression and act as tumor suppressors in many cancers [58–60]. P15, one of the universal CDK inhibitors, can lead to cell cycle halted at G0/G1 checkpoint [61–63]. KLF2, a member of the Kruppel-like factor (KLF) family, also exerts tumor-suppressive roles [64]. P15 and KLF2 have been implicated in various malignancies, including PC [65–70]. We showed here that SNHG15 epression was inversely correlated with that of P15 and KLF2 in PC tissue.

In conclusion, we firstly investigated the expression pattern of SNHG15 in PC tissues and cell lines. SNHG15 may be an indicator of poor prognosis in patients with PC. Additionally, SNHG15 knockdown inhibited PC cell proliferation and tumorigenesis while inducing cell apoptosis. More importantly, there is no study that revealed the molecular mechanism and downstream targets of SNHG15 until now. Here, we demonstrated that SNHG15-mediated oncogenic effect is partly through epigenetically repressing P15 and KLF2 expression. Additional studies are needed to determine whether SNHG15 modulates other targets in PC; however, our findings nonetheless provide novel insight into PC pathogenesis as well as a basis for the improvement of individualized treatment for PC patients.

## MATERIALS AND METHODS

### Tissue samples and cell lines

Forty-eight pancreatic cancer tissues and the corresponding matched non-tumor samples were collected between 2013 and 2016. These patients were performed with surgical resections with signed operation consents in the Second Affiliated Hospital of Nanjing Medical University, and they did not receive any local or systemic treatment before operation. The study design conforms with the regulations of Nanjing Medical University's Ethics Committee. All cell lines used in the study are obtained from American Type Culture Collection (Manassas, VA, USA). The culture condition is set to grow in Dulbecco's modified Eagle's medium (DMEM; Invitrogen, Shanghai, China) with 10% fetal bovine serum (10% FBS) with 5% CO2 in humidified-air at 37 °C

### Total RNA isolation and qRT-PCR assays

The assays of total RNA isolation and qRT-PCR were conducted as previously describled [71]. All the samples are examined three times. The sequences of primers used for the studies are shown in Supplementary Table 1.

### Transfection of PC cell lines

SiRNAs were transfected into PC cell lines using Lipofectamine 2000 (Invitrogen, USA). Plasmid vectors (empty vector and sh-SNHG15) were transfected into PC cell lines using Fugene (Roche, USA). For all sequences, information on si-RNAs and sh-RNAs is listed in Supplementary Table 2. KLF2 and P15 sequences with full length were subcloned into the pcDNA3.1 vector (GENECHEM, Shanghai, China). We adopted qPCR assays to evaluate P15 and KLF2 expression in BxPC-3 cells transfected with pcDNA-P15 or pcDNA-KLF2 and pcDNA3.1 vector was set to a control.

### Cell viability analysis

Cell Proliferation Reagent Kit I (MTT) (Roche Applied Science) and EdU assay kit (Life Technologies Corporation Carlsbad, CA, USA) were used to examine proliferative capacities. Colony formation experiments were performed to monitor PC cells cloning capability.

### Flow cytometry

Si-NC-transfected or siRNA-transfected PC cells were collected after 48h. After staining with PI by CycleTESTTM PLUS DNA Reagent Kit (BD Biosciences), a flow cytometer (FACScan^®^; BDBiosciences) was used to analyze cells. The cell-cycle results elucidate the exact distribution of cells in G0-G1, S, and G2-M phases. Cells for apoptosis analysis were processed with FITC-Annexin-V and propidium iodide (PI) and then FACScan^®^ was used to discriminate cells into viable cells, dead cells, early apoptotic cells, or late apoptotic cells.

### TUNEL staining assay

TUNEL staining assay was performed as previously reported [12]. All experiments were performed in triplicate.

### Subcellular fractionation location

Based on the protocol of manufacturer, PARIS Kit (Life Technologies) was used to separate the nuclear part and cytosolic part in PC cell lines.

### Western blot assay

12% sodium dodecyl sulfate-polyacrylamide gel electrophoresis (SDS-PAGE) was used to separate protein lysates. Then, the protein lysates were transferred to 0.22 mm nitrocellulose membranes (Sigma) with particular antibodies incubation. Antibodies aganist CDK2, CDK4, cleaved caspase-3, and cleaved caspase-9 were supplied by Cell Signaling Technology, Inc. (CST). Antibodies aganinst P15 and KLF2 were supplied by Sigma.

### RNA immunoprecipitation (RIP)

RIP assay was used to investigate whether SNHG15 could interact or bind with the potential binding protein (EZH2 and SUZ12) using EZMagna RIP kit (Millipore, Billerica, MA, USA). Antibodies against EZH2 and SUZ12 were from Millipore.

### Chromatin immunoprecipitation (ChIP)

CHIP experiment was conducted using the EZ-CHIP KIT (Millipore, Billerica, MA, USA). The link between DNA and protein was built through PC cells’ incubation with formaldehyde. Anti-EZH2 and anti-H3K27me3 antibodies (Millipore) were used to immunoprecipitate chromatin fragments. Finally, qRT-PCR assays were performed to analyze the precipitated chromatin DNA. The sequences of CHIP primers are shown in Supplementary Table 3.

### *In vivo* tumor formation assay

Male nude mice of 4 weeks old are supplied by Animal Center of the Nanjing Medical University (Nanjing, China). BxPC-3 cells following stable transfection with empty-vector or sh-SNHG15 were colloected and the concentration of resuspended BxPC-3 cells is 2 × 10^7^ cells/mL. Then the suspended cells were injected into either side of the posterior flank of each mouse. The tumor weight and volumes were tested every 4 days. Up to 24 days after injection, the mice were killed. Then, the tumors formed from empty-vector-transfected or sh-SNHG15-transfected BxPC-3 cells were removed from the mice and were kept for weight measuring and immunohistochemistry (IHC). The instruction conforms with regulations of Nanjing Medical University's Animal Ethics Committee.

### Statistical analysis

SPSS software, version 22.0 (SPSS, Chicago, IL, USA) was used to conduct data analysis. The significant differences between different groups are analyzed by t-test or a chi-square test. The level of P value lower than 0.05 was identified to be statistically significant.

## SUPPLEMENTARY MATERIALS FIGURES AND TABLES


