# Supplementary material for: Long non-coding RNA SNHG15 inhibits P15 and KLF2 expression to promote pancreatic cancer proliferation through EZH2-mediated H3K27me3
Source: Oncotarget. 2017 Aug 18;8(48):84153–67. doi: 10.18632/oncotarget.20359 (PMC5663584; doi:10.18632/oncotarget.20359)
Supplement: Supplementary file 1 [file oncotarget-08-84153-s001.pdf]

Long non-coding RNA SNHG15 inhibits P15 and KLF2 expression to promote pancreatic cancer proliferation through EZH2-mediated H3K27me3

SUPPLEMENTARY MATERIALS

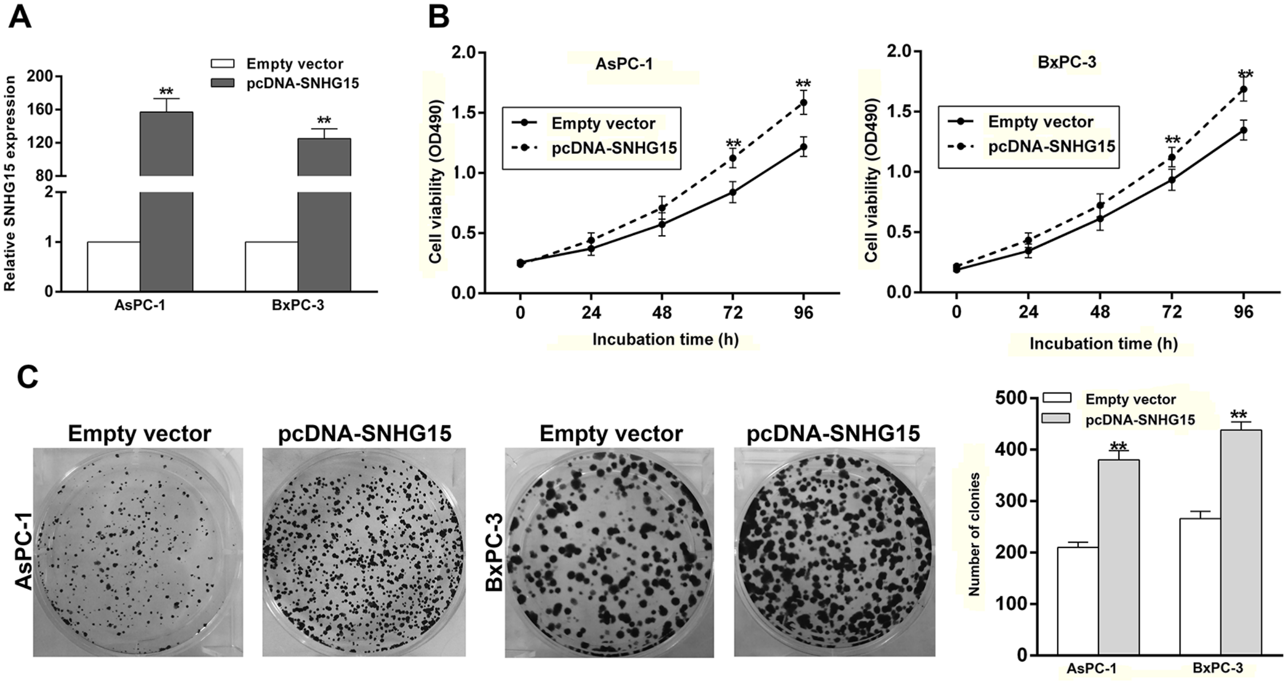

Supplementary Figure 1: SNHG15 overexpression promotes PC cell proliferation *in vitro*. (A) qRT-PCR analysis of SNHG15 expression in AsPC-1 and BxPC-3 cell lines transfected with pCDNA-SNHG15 or the negative control. **(B)** MTT assays were performed to detect the viability of pcDNA-SNHG15 transfected AsPC-1 and BxPC-3 cells. **(C)** Colony-forming growth assays were performed to determine the proliferation of PC cells. The colonies were counted and captured.

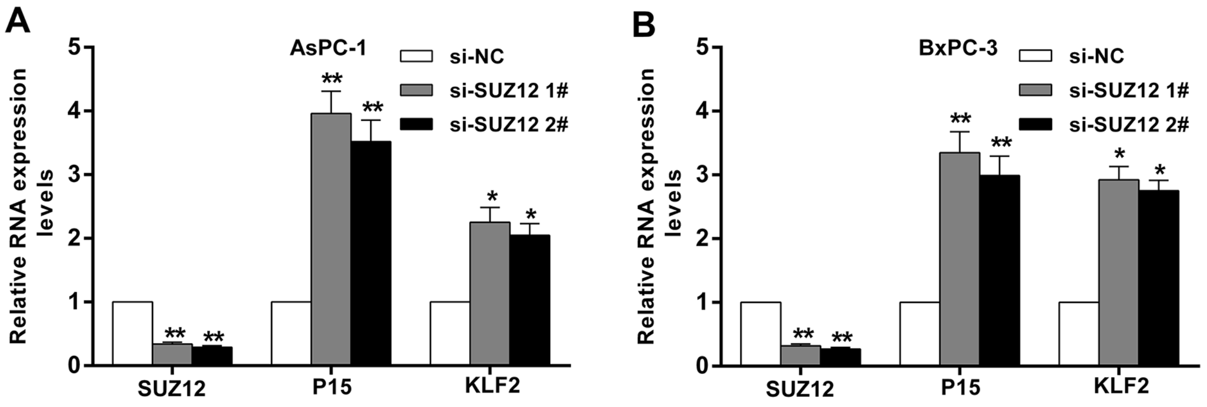

Supplementary Figure 2: The p15 and KLF2 expression levels were determined by qRT-PCR in AsPC-1 and BxPC-3 cells transfected with si-SUZ12 1# or 2#.

Supplementary Table 1: The sequences of qRT-PCR primers

Gene	Forward	Reverse
SNHG15	GCTGAGGTGACGGTCTCAAA	GCCTCCCAGTTTCATGGACA
GAPDH	GAAGAGAGAGACCCTCACGCTG	ACTGTGAGGAGGGGAGATTCAGT
P15	ACGGAGTCAACCGTTTCGGGAG	GGTCGGGTGAGAGTGGCAGG
P16	ATGGAGCCTTCGGCTGACT	GGCCTCCGACCGTAACTATT
P21	CAGCAGAGGAAGACCATGTG	GGCGTTTGGAGTGGTAGAAA
P27	TGCAACCGACGATTCTTCTACTCAA	CAAGCAGTGATGTATCTGATAAACAAGG
P57	CACGATGGAGCGTCTTGTC	CCTGCTGGAAGTCGTAATCC
KLF2	TTCGGTCTCTTCGACGACG	TGCGAACTCTTGGTGTAGGTC
PTEN	AGTTCCCTCAGCCGTTACCT	AGGTTTCCTCTGGTCCTGGT
EZH2	TGCACATCCTGACTTCTGTG	AAGGGCATTCACCAACTCC
U6	CTCGCTTCGGCAGCACA	AACGCTTCACGAATTTGCGT

Supplementary Table 2: The information of siRNAs

SNHG15 1#	UGGAUGACUAAACUGCCGAAGACCG	CGGUCUUCGGCAGUUUAGUCAUCCA
SNHG15 2#	UGUAGAAACACUGACGGAUGGCAGG	CCUGCCAUCCGUCAGUGUUUCUACA
SNHG15 3#	GAGCAAGUUUGAAACCUCGCUUGUU	AACAAGCGAGGUUUCAAACUUCCUC
si-EZH2 1#	GAGGUUCAGACGAGCUGAUUU	
si-EZH2 2#	GCUCCUCUAACCAUGUUUATT	

Supplementary Table 3: The primers in CHIP assays

	Forward	Reverse
P15 P	TTTGGCCTCCTCCCCAAATG	ACGGCAGATGGGAATTCGTT
KLF2 P	ACGGGCTTATTGAGGTTGG	GCCTGGGTGACAGAGGAGAC
